# Supplementary material for: Thermochemical Characterization of Eight Seaweed Species and Evaluation of Their Potential Use as an Alternative for Biofuel Production and Source of Bioactive Compounds
Source: Int J Mol Sci. 2022 Feb 21;23(4):2355. doi: 10.3390/ijms23042355 (PMC8880020; doi:10.3390/ijms23042355)
Supplement: Supplementary file 1 [file ijms-23-02355-s001.zip › ijms-1561434-supplementary.pdf]

**SUPPLEMENTARY MATERIAL**

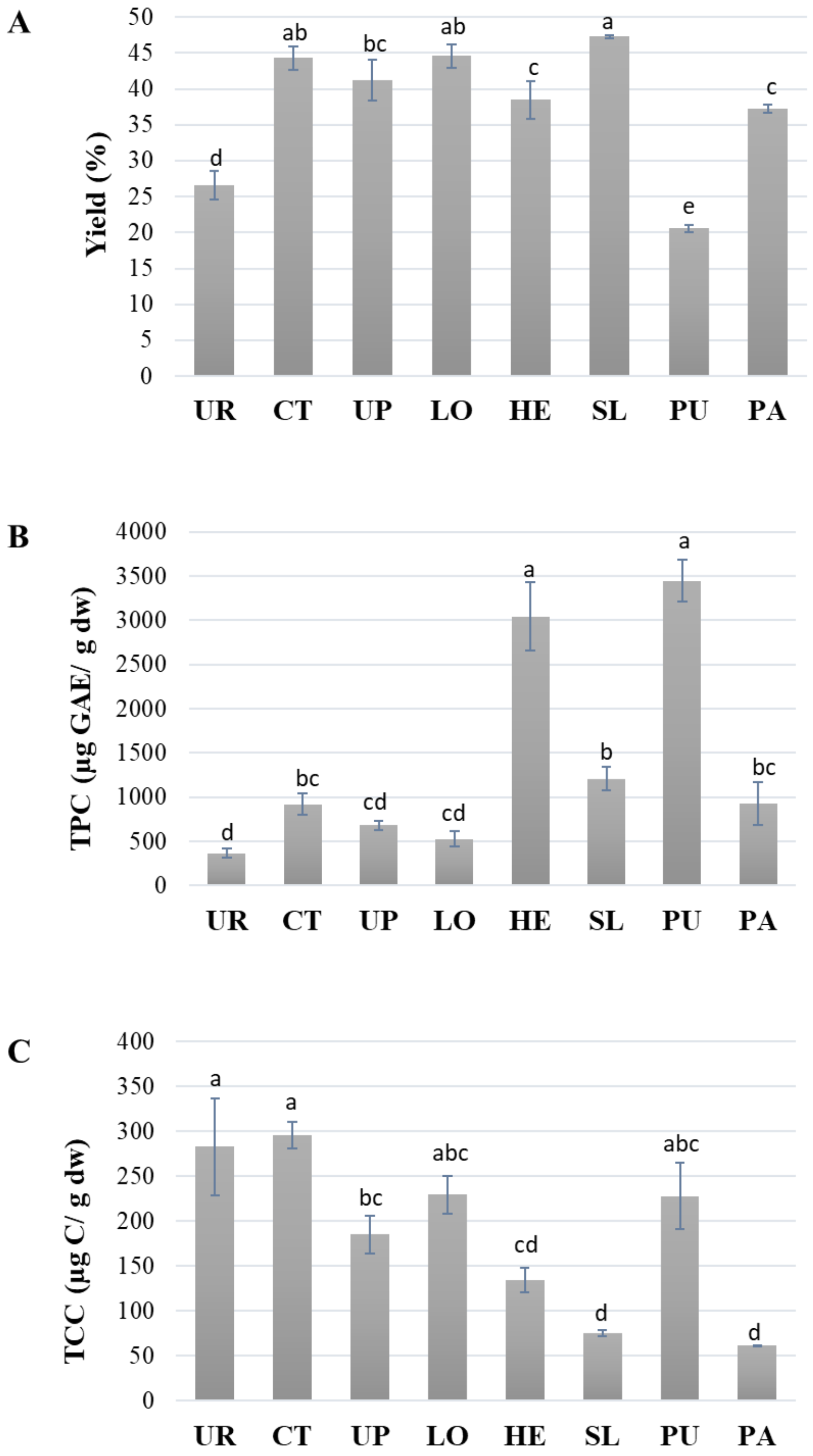


**Figure S1**. (**A**) Extraction yield, (**B**) total phenolic content (TPC) and (**C**) total carotenoid content (TCC) of the studied seaweeds. **GAE**: Gallic acid equivalents; **C**: Carotenoids. **UR**: *Ulva rigida*, **CT**: *Codium tomentosum*, **UP**: *Undaria pinnatifida*, **LO**: *Laminaria ochroleuca*, **HE**: *Himanthalia elongata*, **SL**: *Saccharina latissima*, **PU**: *Porphyra purpurea*, **PA**: *Palmaria palmata*.

**Table S2**. Regression parameters and determination coefficients for adjusted data from DPPH and ABTS assays corresponding to each studied seaweed.

| SEAWEED | MODEL PARAMETERS | | $R^2$ | REGRESSION |
|---|---|---|---|---|
| | a | b | | |
| ***DPPH*** | | | | |
| ***HE*** | 67.263 | 8.0452 | 0.9576 | Linear |
| ***ABTS*** | | | | |
| ***UR*** | 9.9334 | 33.557 | 0.9105 | Non-linear |
| ***CT*** | 41.743 | 19.758 | 0.9915 | Linear |
| ***UP*** | 13.033 | 70.458 | 0.9783 | Non-linear |
| ***LO*** | 12.372 | 87.236 | 0.9513 | Non-linear |
| ***HE*** | 18.284 | 97.033 | 0.9331 | Non-linear |
| ***SL*** | 17.265 | 84.071 | 0.9227 | Non-linear |
| ***PU*** | 13.805 | 56.334 | 0.9736 | Non-linear |
| ***PA*** | 10.034 | 65.765 | 0.0866 | Non-linear |

***UR***, *Ulva rigida;* ***CT***, *Codium tomentosum;* ***UP***, *Undaria pinnatifida;* ***LO***, *Laminaria ochroleuca;* ***HE***, *Himanthalia elongata;* ***SL***, *Saccharina latissima;* ***PU***, *Porphyra purpurea;* ***P****A, Palmaria palmata.*
